# Working Memory Training in Patients with Chronic Schizophrenia: A Pilot Study

**DOI:** 10.1155/2013/154867

**Published:** 2013-02-26

**Authors:** Martina Hubacher, Marcus Weiland, Pasquale Calabrese, Gabriela Stoppe, Markus Stöcklin, David Fischer-Barnicol, Klaus Opwis, Iris-Katharina Penner

**Affiliations:** ^1^Department of Cognitive Psychology and Methodology, University of Basel, Missionstrasse 60/62, 4055 Basel, Switzerland; ^2^Department of General Psychiatry, University Psychiatric Hospitals, Wilhelm Klein-Strasse 27, 4012 Basel, Switzerland; ^3^Division of Molecular and Cognitive Neuroscience, University of Basel, Switzerland

## Abstract

*Background*. There is evidence that patients with schizophrenia suffer from decline in working memory performance with consequences for psychosocial outcome. *Objective*. To evaluate the efficacy of a computerized working memory training program (*BrainStim*) in patients with chronic schizophrenia. *Methods*. Twenty-nine inpatients with chronic schizophrenia were assigned to either the intervention group receiving working memory training (*N* = 15) or the control group without intervention (*N* = 14). Training was performed four times a week for 45 minutes during four weeks under neuropsychological supervision. At baseline and followup all participants underwent neuropsychological testing. *Results*. Pre-post comparisons of neuropsychological measures showed improvements in visual and verbal working memories and visual short-term memory with small and large effect sizes in the intervention group. In contrast, the control group showed decreased performance in verbal working memory and only slight changes in visual working memory and visual and verbal short-term memories after 4 weeks. Analyses of training profiles during application of *BrainStim* revealed increased performance over the 4-week training period. *Conclusions*. The applied training tool *BrainStim* improved working memory and short-term memory in patients with chronic schizophrenia. The present study implies that chronic schizophrenic patients can benefit from computerized cognitive remediation training of working memory in a clinical setting.

## 1. Introduction

Cognitive dysfunctions are known to be a core feature of schizophrenia. Nearly 95% of all schizophrenic patients are impaired in cognitive functioning, 65% of the patients show deficits in cognitive flexibility, 75% show poorer performance in planning tasks, and 65% show deficits in working memory [1]. These deficits appear early in the disease course and might exist before the first positive or even negative symptoms become manifested [2]. Additionally, cognitive performance appears to be a possible predictor for remission [3]. The significance of impaired cognitive functioning in schizophrenia is highlighted by the discussion about the inclusion of cognitive impairment in the DSM criteria [4, 5].

Among cognitive functions, working memory is fundamental. This function includes attending to current events, maintaining and manipulating incoming information, and integrating them into long-term memory. Working memory actively processes and stores information [6]. In the original model proposed by Baddeley and Hitch [7], working memory contains three subsystems, a *phonological loop,* and a *visuospatial sketchpad*, which represent the two modality-specific slave systems. These two slave systems are controlled and regulated by the *central executive*. In a revised model, Baddeley [8] added the *episodic buffer*, a multimodal storage system with limited capacity that supports the exchange between the first two slave systems. Due to the functions of working memory, and its impact as interface between perception, long-term memory and higher cognitive functions, it is likely that malfunctioning highly affects everyday life. Since, in schizophrenic patients, working memory deficits can be observed in all three traditional subsystems [9, 10] it is not astonishing that a wide range of behaviours is affected [11]. Although antipsychotic medication ameliorates positive and negative symptoms of the disease, unfortunately, the cognitive problems do not seem to improve [12–14] and are described to be stable over time [15]. Thus, alternative approaches able to improve cognitive functioning per se and the important domain working memory in particular are warranted. 

Although some studies reported failure to generalize beyond practice effects (e.g., [16, 17]), there is a tantamount of evidence that cognitive training might be beneficial in patients with schizophrenia. Further, it seems to be the best-supported approach to treat cognitive impairment [18]. A meta-analysis by McGurk et al. [19] including 26 studies with computerized and noncomputerized trainings reports a medium effect size on cognitive functioning after remediation training, as well as psychosocial functioning, and a small effect on clinical symptoms. Cavallaro and colleagues [20] showed the effectiveness of computer-aided remediation on several cognitive domains. A meta-analysis by Grynszpan et al. [21] confirms the moderate effect of computerized training in schizophrenia. Especially in working memory, improvements are reported after a combination of work-therapy and neurocognitive enhancement therapy [22, 23], as well as after computer-assisted cognitive remediation [24, 25]. A recent meta-analysis composing 40 studies on the efficacy of cognitive remediation in schizophrenia [26] confirms that patients profit from this kind of intervention. Effect sizes on the posttreatment cognitive outcomes over all studies were described to be small to moderate. 

Moreover, though some of the studies tackled working memory capacity, the tests and their presentation mode administered as part of the exercises were heterogeneous. To follow our hypothesis of working memory being one of the central components for efficient cognitive functioning we applied a computerized tool called *BrainStim* to inpatients with chronic schizophrenia and compared their posttreatment cognitive performance to those of an untrained control group. The training tool *BrainStim* has already been evaluated in a pilot study on healthy elderly people [27] and on patients with multiple sclerosis [28, 29]. In both studies, positive effects were especially found for working memory performance, while transfer effects to other cognitive domains turned out to be rather small. 

 Thus, our hypotheses for the present study were that (a) inpatients with chronic schizophrenia in the treatment group would benefit from training by improving their working memory performance, while performance of untrained patients was assumed to remain stable, and (b) increased performance within the training as an indicator of learning capability can be shown in patients with chronic schizophrenia.

## 2. Method

### 2.1. Participants

Forty-four inpatients from the University Psychiatric Hospitals in Basel, Switzerland, were recruited. All participants had a definite diagnosis of schizophrenia according to ICD-10 and were treated as inpatients for treatment of exacerbation of their disorder and/or for (long term) rehabilitation. At the time of study enclosure they were all stable since more than two weeks (based on evaluation of treating psychiatrist). 23 patients were included in the training group, whereas the other 21 participants were put on a waiting list and served as control group with the opportunity to complete the training after one month. 7 patients of the intervention group (30%) quit the training due to their discharge from the hospital, and one more had to be excluded due to a lack of compliance in the posttest. In the control group, 7 patients (33%) quit because of discharge from the hospital. The remaining 15 patients in the training group were all diagnosed with paranoid schizophrenia (F20.0). In the control group (*N* = 14) there was one patient with a hebephrenic schizophrenia (F20.1) as well as one patient with an undifferentiated type of schizophrenia (F20.3); the other 12 participants all had a diagnosis of paranoid schizophrenia (F20.0). Details on medication are shown in Table 1. All patients gave written informed consent to participate in the study, which was approved by the local ethics committee of the University of Basel, Switzerland.

### 2.2. Procedure

All participants were examined twice by a neuropsychological testbattery the first time to assess baseline performance and the second time to evaluate posttreatment effects. After baseline testing the intervention group received the training by means of the *BrainStim* software [30] during four weeks, four times a week for 45 minutes, supervised by a master student in psychology, whereas the control group solely received routine clinical treatment. The efficacy of the treatment schedule was verified in a former study by Vogt et al. [28]. 

### 2.3. Training Tool *BrainStim*

*BrainStim* is a computerized program to improve working memory performance. It contains three different modules: two focusing on the spatial aspects of working memory and the third on the verbal aspects. The first module, called *City Map*, trains spatial orientation. Participants have to memorize either a visually or verbally presented route. Afterwards, the route has to be reconstructed from memory on a virtual city map. The module *Find Pairs *trains visual memory. Patients have to remember the location of two similar turned over cards. The last module, *Memorize Numbers*, presents numbers for a short period of time, and patients are asked to recall them, after solving an arithmetic distraction task. This module focuses on verbal working memory and the central executive. All modules automatically adapt their level of difficulty according to the performance of the user. After several correct answers, the level increases to a higher degree, whereas after a certain number of incorrect answers the user was forced to repeat the previous level. With each added level the amount of information to be memorized increases. *BrainStim* stores all training data in logfiles for further analysis.

### 2.4. Applied Neuropsychological Measures

During baseline assessment, depression was measured with the German version of the Center for Epidemiologic Studies Depression Scale (CES-D [31]; Allgemeine Depressionsskala, ADS [32]), and the premorbid intellectual level was assessed with a German multiple-choice vocabulary IQ test, MWT-A [33]. The neuropsychological test battery included the following tests: *verbal fluency test* measuring executive functions and mental speed; *selective reminding test* assessing verbal short- and long-term memory by means of three measures: long-term retrieval, consistent long-term retrieval, and delayed recall; 10/36* spatial recall* test measuring visual short- and long-term memory by an immediate and a delayed recall version; *Symbol Digit Modalities Test* (*SDMT*) to test for information processing speed and working memory; and *1-back task*, adapted from the Test Battery for Attention Performance (TAP [34]) to test for short-term memory and attention. For short-term memory we additionally applied the *Corsi Blocks Forward* and the *Digit Span Forward* from the Wechsler Memory Scale revised (WMS-R [35]). Working memory, the domain of major interest in our study, was measured with the *Paced Auditory Serial Addition Test *(*PASAT*)[36], *the 2-back and 3-back task*, adapted from the TAP [34], the *Corsi Blocks backward,* and the *Digit Span Backward* from the WMS-R [35]. Completion of the test battery took approximately 75 to 90 minutes.

Taking into account the already proven specificity of *BrainStim* on working memory performance in previous studies [27, 28], to disburden the patients and to hereby improve adherence until the end of the study, only the following tests were applied for the posttreatment assessment: the *PASAT*, the 1-back, 2-back and 3-back tasks, the *Corsi Blocks* Forward and Backward as well as the *Digit Span* task Forward and Backward. This posttreatment assessment took approximately 30 minutes to be completed.

### 2.5. Statistics

To evaluate cognitive performance, *z* values corrected for age were calculated. Patients cognitive performance was classified as reduced if they reached a *z* value smaller than −1.65 since this cutoff represents the ninetieth confidence interval. About one-third of the participants could not complete the *PASAT*, 1-back and/or 2-back tasks and more than three-quarters could not complete the 3-back task. Therefore these tests were excluded from further analyses. Due to the small sample sizes, comparison between groups on cognitive and psychosocial variables was performed using the nonparametric Mann-Whitney *U* Test.

 The log files of the training tool *BrainStim* were read out, and average levels of difficulty from first and last training were compared. It has to be noted that not all patients were able to complete all 16 training sessions (11 patients completed all sessions and 4 completed only 15 sessions), therefore; analysis was computed by means of the last observation carried forward method. To check whether the improvement of performance was significant, the averaged levels of difficulty from the first and the last available training were compared using the nonparametric Wilcoxon signed-ranks test (exact). This nonparametric test was also chosen due to the small sample size.

To quantify the comparison between pre- and posttest performance, *z* value differences between pre- and posttest were calculated for both participant groups. Mann-Whitney *U* Test was used to evaluate these differences between the two groups. Further, Cohen's *d* for effect sizes was calculated [37]. Therefore, the mean differences between the training group [38] and the control group (CG) were divided by the pooled standard deviation (*d* = (*M*_TG_ − *M*_CG_)/√((SD_TG_^2^∗(*N*_TG_ − 1)+  SD_CG_^2^∗(*N*_CG_ − 1))/(*N*_TG_ + *N*_CG_ − 2))). To evaluate the size of the effect, *d* = .2 was rated as a small effect, *d* = .5 as a moderate, and *d* = .8 as a large effect.

## 3. Results

### 3.1. Participants

As displayed in Table 1, the 15 patients (women: 6; men: 9) in the training group and the 14 patients (women: 8; men: 6) in the control group showed no significant differences with regard to gender (*χ* ^2^(1) = .852, *P* = .466), education, age, severity of illness (rating based on the clinical experience of the treating psychiatrist), and duration of illness and depression. The control group seemed to have a slightly higher intellectual level. 

All patients showed rather poor cognitive performance. In the training group 12 out of 15 patients (80%) showed reduced performance in one or more cognitive domains, and in the control group 13 of 14 (93%) showed impaired cognition. However, cognitive profiles did not differ between the intervention and control groups at baseline, except for mental speed. 

Mean depression score was below the cutoff value (>23) in both groups. When analysing individual data, 6 out of 15 patients (40%) in the intervention group and 5 out of 14 patients (36%) in the control group had a total score above the cutoff, indicating comorbid depression.

### 3.2. Training Tool *BrainStim*

In our group of 15 patients, who received the training, 11 patients completed all 16 sessions of training, while 4 completed only 15 sessions.

**Figure 1:**
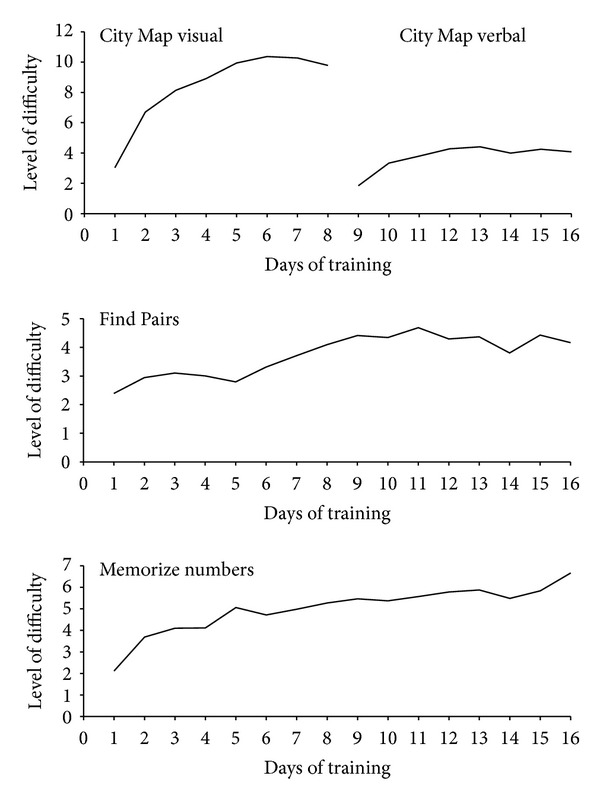
Averaged performance during training with *BrainStim* for the module City Map visual and verbal, the module Find Pairs, and the module Memorize numbers (*N* = 15).

Figure 1 illustrates the averaged training performance in the three modules. In all three modules the patients were able to increase the level of task difficulty during the four weeks of training. With training, the curve of *City Map visual* seems to approach asymptotically the highest level, whereas progress in the other modules is considerably smaller.

Means and standard deviations of levels of difficulty for the first and last training are displayed in Table 2. In all three modules participants managed to significantly increase their performance: *City Map* with visual instructions (*z* = 3.41, *P* < .001), *City Map* with verbal instructions (*z* = 3.29, *P* = .001), *Find Pairs* (*z* = 3.07, *P* = .002), and *Memorize Numbers* (*z* = 3.41, *P* = .001). 

### 3.3. Neuropsychological Test Battery

**Figure 2:**
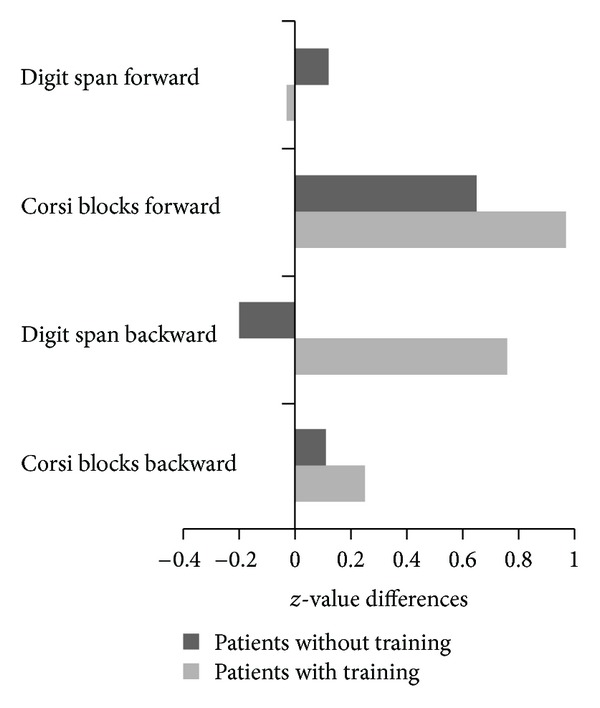
Differences in *z* values between pre- and posttesting. Negative values imply a decrease in performance; positive values demonstrate an increase.

Means and standard deviations for each test, effect sizes and results of the Mann-Whitney *U* Test are displayed in Table 3, and changes in *z* values of cognitive performance are shown in Figure 2. In verbal short-term memory (*Digit Span Forward*), both groups remained stable in their performance. In visual short-term memory (*Corsi Blocks Forward)* both groups showed an increased outcome after four weeks. Patients with training increased more than patients in the control group. Although this difference was not statistically significant, Cohen's *d* implied a relevant effect. The group without training decreased in verbal working memory performance (*Digit Span Backward*) whereas the trained group improved. Here, a significant difference between the two groups was found and Cohen's *d* indicated a large effect. In visual working memory performance (*Corsi Blocks Backward*) both groups improved slightly, but no statistically significant difference was detectable between the two groups.

## 4. Discussion

In the present study, the effectiveness of a computerized remediation training for working memory was evaluated in patients with chronic schizophrenia. During the training itself, patients showed improved performance when comparing the lasttioniry to the first training sessions in all three modules indicating learning potential. 

Concerning the postintervention effects on the neuropsychological outcomes no effects were found for verbal short-term memory. The training showed no differential effect on this cognitive domain, confirming our expectations. This result is in accordance with Klingberg et al. [39], who did not find changes in verbal short-term memory in the stabilization phase of schizophrenia. Further, a small but not statistically significant effect was found on visual short-term memory. Trained patients improved more than untrained subjects. The improvement detected in both groups leads to the conclusion that this domain may recover in the stabilization phase after an acute schizophrenic episode, but that recovery can be supported and increased by a cognitive training. 

In verbal working memory, a large effect was found. Patients who received the training showed an increased performance after four weeks, whereas patients without training decreased. Our findings on verbal working memory are in accordance with results by McGurk et al. [19] who reported medium effect sizes for this cognitive domain. In visual working memory patients with training improved more than patients without. Although this effect was not statistically significant it goes into the expected direction.

In contrast to the results in our study, several studies recently showed no effectiveness of their computer-assisted cognitive remediation therapy on most cognitive functions, including working memory [16, 40, 41]. In a most recent study by Rass and coworkers [17] the authors were not able to show a generalized effect on the basis of a computer-assisted training programme. However, the training sessions were administered biweekly for two hours, each indicates that not a massed training was administered. Most importantly, their groups showed no systematic variation between treatment and comparison groups. Thus, an increase in sample size would have led to a statistical difference hardly. In contrast, our training sessions were scheduled four times a week for 45 minutes each, and our groups showed a systematic direction towards a better outcome for the intervention group. Cavallaro et al. [20] reported effects on several cognitive domains but not on working memory. A possible explanation for this discrepancy may be due to the content of training. Previous studies focused on other cognitive domains then working memory and trained according to other treatment regimen. Thus, not every training seems to provoke beneficial effects meaning that content and modality of training may be more important than what has been formerly assumed. Further studies should compare different treatment programs to investigate the mechanisms of cognitive intervention and to finally specify a gold-standard for cognitive remediation in schizophrenia.

An additional next step would be to study the underlying neural mechanisms of cognitive remediation in schizophrenia. Haut et al. [42] reported increased activation in attention and working memory networks after a training designed to train these cognitive functions. Subramaniam and colleagues [43] recently described that improvements in reality monitoring correlated with medial prefrontal cortex activity after training.

Despite the fact that our hypotheses were corroborated, the present pilot study has several limitations, which have to be considered. First, even if the characteristics of the two groups of patients at baseline did not differ systematically, they differed in terms of premorbid intellectual level, in that the control group had higher measures, and in terms of executive functioning, in that the intervention group scored higher. This might have influenced the effects of our training. A higher intellectual level and thus a higher cognitive brain reserve as well as higher scores in executive functioning may support effectiveness of such an intervention.

Second, symptom severity was only rated by treating psychiatrists at baseline but not after the cognitive intervention. Thus we cannot relate changes in symptom severity to improvements in working memory after 4 weeks.

Third, only four cognitive outcome measures could finally be included into the statistical analyses since most participants could not complete the more complex tasks such as the n-back and PASAT. This demonstrates the degree of impairment in this patient cohort and the need for specified additional cognitive treatment approaches. Of interest is that after the training more patients were finally be able to complete these tasks, and they reported to feel more confident to do so. Although the observed changes cannot be rated statistically, they are certainly of relevance for patients. Fourth, the drop-out rate was high. One third of patients did not complete the study. All of them refused to return to the clinic for the training or neuropsychological posttesting after their discharge. In contrast to other studies, participants in this study got no financial compensation, which might be reflected by the high drop-out rate. However, a design without any financial incentive represents rather a daily clinical routine and is, therefore, more appropriate in predicting training success in this setting. Fifth, to overcome patients' adherence problem we decided to shorten the posttraining neuropsychological battery and to solely focus on working memory outcomes. By this approach, transfer effects of training could not be assessed. Sixth, the training was supervised one-by-one. Patients often reported that they enjoyed the additional advertence. Therefore, it is likely that at least a part of the improvement might be due to this additional advertence and the related additional effort the patients were willing to invest. Unfortunately, the present design does not allow separating the effects of training from the possible effects of additional advertence and motivation. We would suggest including an active control group additionally to the treatment-as-usual group for further studies. Seventh, patients were assigned to the treatment or waiting control groups consecutively by their inclusion in the study the (first 15 patients were assigned to the treatment group, and the following 14 patients were assigned to the waiting control group) meaning that no real randomisation took place. Eightly, a follow-up testing to survey long-term effects of cognitive remediation is missing. Wykes et al. [44] reported increased working memory performance, compared to baseline, 6 months after the end of their cognitive remediation training. Though the persistence of cognitive remediation has already been described in other studies we cannot assume that follow-up effects would also have been detectable in our study. Follow-up measurements necessarily have to be included in further studies to address this important topic.

A last limitation to discuss is the small sample size of the two groups. The found effects should be verified in a larger sample. In a larger cohort the different improvement of the two groups in visual working memory might even reach statistical significance. Further, effect sizes were calculated to quantify the changes in cognitive performance. Wykes and Huddy [45] discuss that size of effect does not necessarily matter. They show that improvement in cognition may have no effect on functioning. The present study includes no scales measuring general functioning or factors of daily living. Therefore, a transfer of better cognitive abilities into work rehabilitation and quality of life cannot be evaluated, but it can be presumed from other studies that there might be an effect [46, 47]. One study showed that especially auditory attention and working memory improvement predict changes in life skills after cognitive remediation therapy [48]. Cognitive outcome influences negative symptoms [49], what in turn affects the daily outcome [50]. Actually, Lecardeur et al. [51] even report an influence on psychotic symptoms.

Despite the unidirectional variation between the groups, showing a clear advantage for the training group, medication together with illness severity and chronicity might have attenuated our results. Thus, explanatory power of this study is restricted due to its limitations, and generalisation of the results should be regarded with caution. Nevertheless, this study clearly shows beneficial effects even in patients with chronic schizophrenia. Additional to the statistical proofs, patients reported to have enjoyed training, that they could transfer learned strategies to everyday life, and that they wished to work with a computer in the future, since for several participants it was the first time to work with computers. A couple of patients took the opportunity to work with another computerized training in occupational therapy. This feedback is consistent with the one reported by Bender et al. [52]. Combined with other interventions cognitive trainings seem to be more effective [26], and the effects of intervention are more durable [53]. Therefore, in sum, cognitive training seems to be a valuable additional treatment approach for schizophrenic patients in clinical everyday life, besides the usual medication-based treatments.

## Figures and Tables

**Table 1:** Demographic, clinical, and baseline characteristics of participants and percentage of patients with reduced cognitive performance.

	Intervention group (*N* = 15)		Control group (*N* = 14)		
Demographic and clinical data	*M*	SD	*M*	SD	*P* value

Age^a^	39.53	10.78	44.36	13.47	.33
Education^a,b^	0.87	0.64	0.86	0.86	.91
Severity of illness^a,c^	3.60	1.60	3.64	1.08	.81
Duration of illness^a,d^	6.27	5.64	11.83	7.28	.12
Depression score^a^					
ADS-L	22.93	9.15	21.29	7.14	.62
Intellectual level^a,e^					
MTW-A	25.73	4.17	29.17	4.91	.03

Cognition^f^	*M*	SD (%)	*M*	SD (%)	*P* value

Verbal memory^a^					
Selective reminding LTR^g^	−1.77	1.87 (47)	−1.89	1.60 (46)	.72
Selective reminding consistent LTR^g^	−1.54	1.43 (47)	−1.76	1.05 (54)	.56
Selective reminding delayed recall	−1.41	1.51 (47)	−1.43	1.35 (31)	.93
Visual memory^a^					
Spatial recall	−0.91	0.85 (27)	−0.86	1.05 (21)	.81
Spatial recall delayed recall	−0.75	1.24 (20)	−0.62	1.24 (29)	.81
Processing speed^a^					
SDMT	−1.52	1.09 (53)	−1.66	1.49 (57)	.85
Executive functioning^a^					
Verbal fluency	−1.30	0.51 (20)	−2.09	0.84 (57)	<.01

Medication types		*n *		*n *	

No medications		2		2	
Atypical antipsychotics		13		10	
Conventional antipsychotics		2		3	
Antidepressants		4		2	
Anticonvulsants		2		1	
Anticholinergics		5		4	
Other psychoactive medication		4		2	

^a^Mann-Whitney * U* Test; ^b^education: 0: secondary school, 1: college, 2: university; ^c^1: mild, 3: moderate, 5: severe; ^d^data only available for 11 patients in the intervention group and 6 patients in the control group; ^e^data only available for 12 of the 14 patients in the control group; ^f^displayed are mean *z* values; ^g^LTR: long-term retrieval.

**Table 2:** Mean levels of difficulty and standard deviations from the first training and the last training of the modules used for training (*N* = 15).

	First training		Last training	
	*M*	SD	*M*	SD
City Map visual	3.03	1.37	10.10	2.74
City Map verbal	1.85	0.48	4.49	2.09
Find Pairs	2.39	0.49	4.90	3.20
Memorize numbers	2.22	1.10	6.22	3.68

**Table 3:** Means and standard deviations of *z* values for intervention group and control group and effect sizes and results of the comparison (Mann-Whitney *U* Test) of *z* value differences of pre- and posttests between the two groups.

		Patient group with training (*n* = 15)				Patient group without training (*n* = 14)						
Neuropsychological assessment		Baseline		After 4 weeks		Baseline		After 4 weeks		*d*	*z*	*P*
		*M*	SD	*M*	SD	*M*	SD	*M*	SD			
Short term memory	verbal^a^	−1.32	0.95	−1.35	0.79	−0.71	0.87	−0.59	1.04	0.19	−0.71	.50
	visual^b^	−1.02	0.96	−0.05	1.17	−0.95	1.13	−0.30	1.72	0.21	−0.04	.98
Working memory	verbal^c^	−1.18	1.17	−0.42	1.31	−0.89	0.91	−1.09	0.77	1.04	−2.37	.02
	visual^d ^	−0.76	1.11	−0.51	1.18	−1.50	1.25	−1.39	1.60	0.11	−0.49	.65

^a^Digit span forward,^ b^Corsi blocks forward, ^c^Digit span backward,^ d^Corsi blocks backward.
